# Supplementary figures and images for: A Simple Negative Interaction in the Positive Transcriptional Feedback of a Single Gene Is Sufficient to Produce Reliable Oscillations
Source: PLoS One. 2011 Nov 10;6(11):e27414. doi: 10.1371/journal.pone.0027414 (PMC3244268; doi:10.1371/journal.pone.0027414)

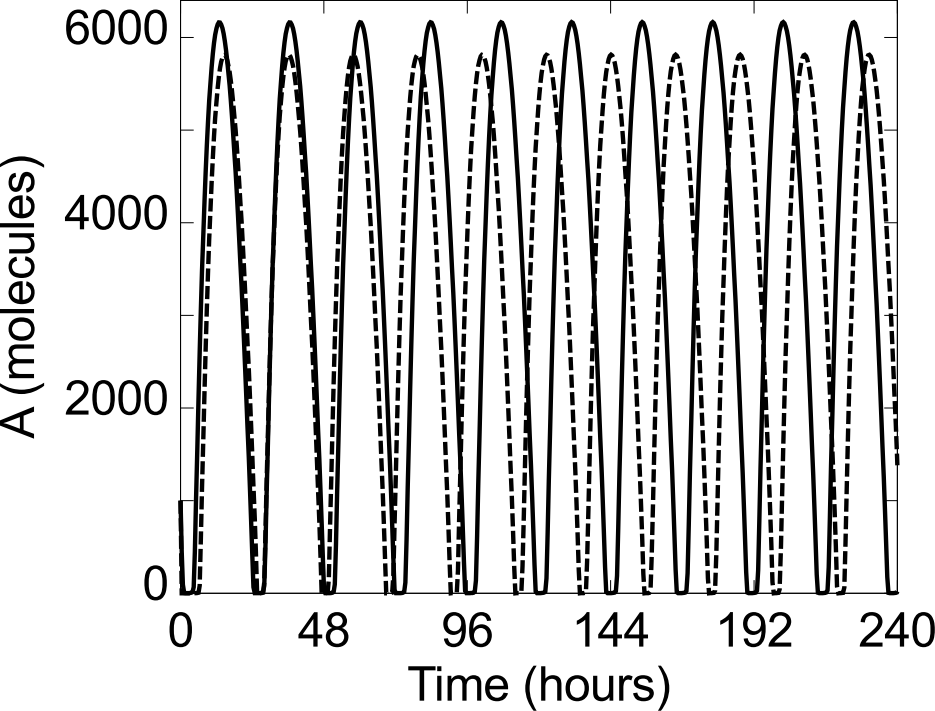

Supplement: Figure S1 — Time evolution of with and without a low number molecules. Comparison between deterministic simulation of the time evolution of with (dashed line) and without (solid line) a low number of , , and molecules. (Solid line graph: the values of the parameters are as in the section Methods: Biochemical reactions and rates. Dashed line graph: the changed rates are hour, hour, molecules hour, hour and molecules hour.) (PDF) [file pone.0027414.s001.pdf]

**A**

Counts for 1,000 cycles

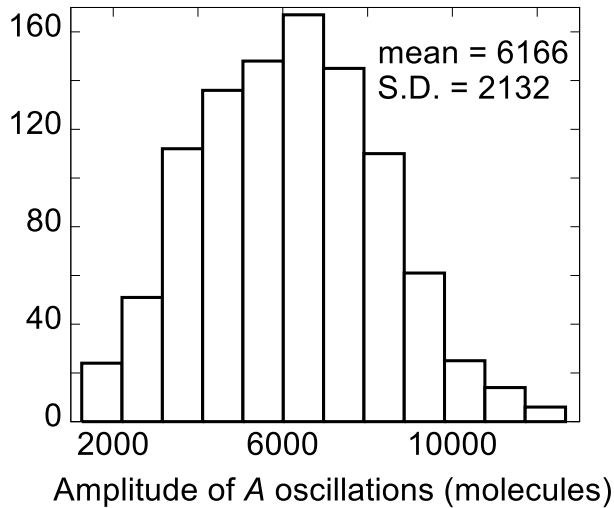**B**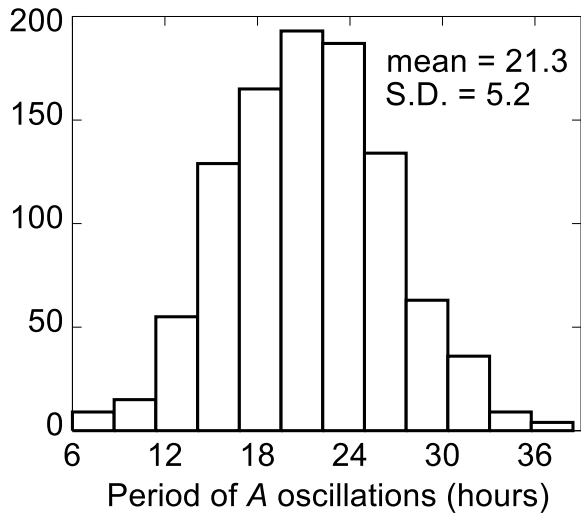

Supplement: Figure S2 — Amplitude and period histograms of the stochastic simulation of . A, B. Amplitude and period histograms of the stochastic simulation of , respectively. The values of the parameters are as in the section Methods: Biochemical reactions and rates but now we set hour, hour, molecules hour, hour and molecules hour. (A and B were calculated for 1,000 successive cycles. We assumed that a cycle occurs if the number of proteins increases to 1,000 molecules and then decreases to 700 molecules. The amplitude was calculated as the greatest number of molecules in each cycle. The period was calculated as the time interval that it takes the numbers of proteins to reach 1,000 molecules for the first time in two successive cycles.) (PDF) [file pone.0027414.s002.pdf]

**A**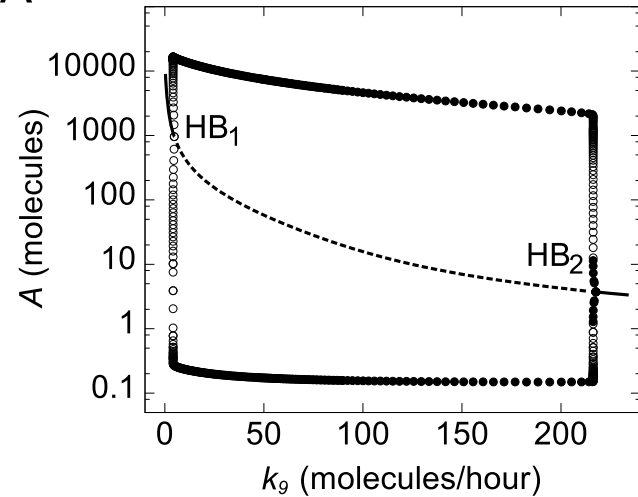**B**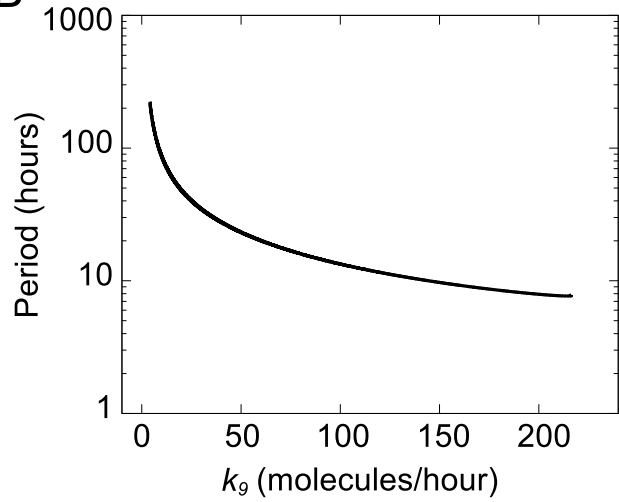

Supplement: Figure S3 — Bifurcation diagram of the reduced model. A. Bifurcation diagram depending on . The solid/dashed line represents stable/unstable fixed points. Black/white circles are the maximum and minimum values of during unstable/stable oscillations. HB denotes a Hopf Bifurcation point. HB and HB appear when the value of is 4.78 and 217.6 molecules hour, respectively. B. Period of the stable oscillations in A. (PDF) [file pone.0027414.s003.pdf]

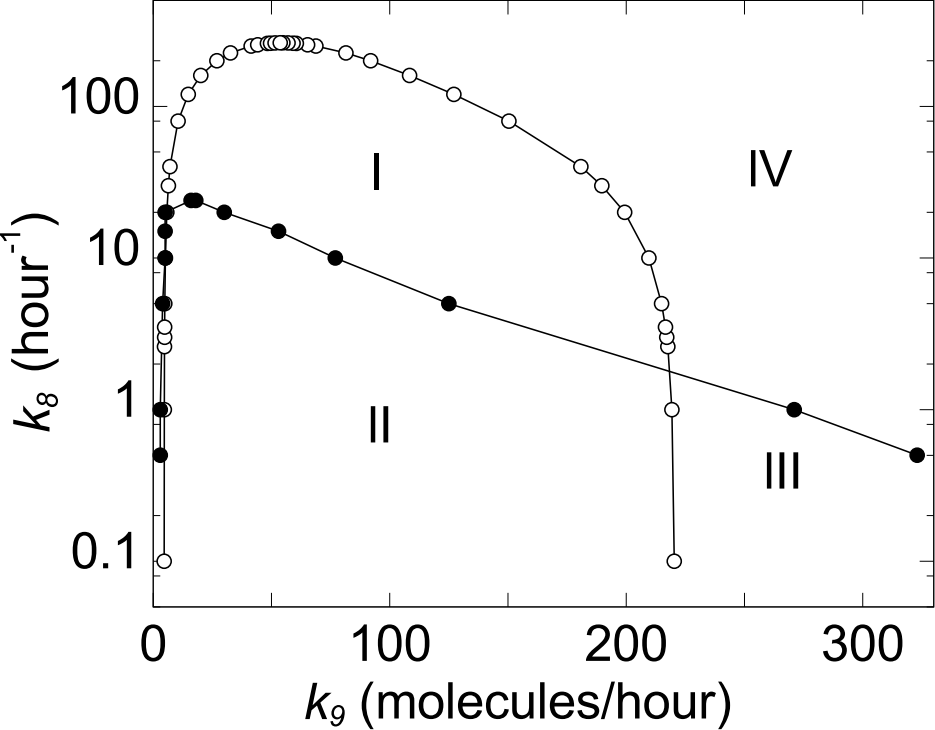

Supplement: Figure S4 — Oscillatory regions in the reduced and stochastic models depending on and . Region I. Oscillations in reduced model. Region II. Oscillations in both reduced and stochastic model. Region III. Oscillations in the stochastic model. Region IV. No oscillations in any model. White circles represent the locus of Hopf bifurcations in the reduced model (data are presented in Table S2). Black circles represent locus of oscillations in the stochastic simulation (data are presented in Table S3). We assumed in the stochastic case that oscillations occur in a region if the correlation in the first period is greater than 0.2. (The lines connecting circles are designed to clearly single out the different regions.) (PDF) [file pone.0027414.s004.pdf]

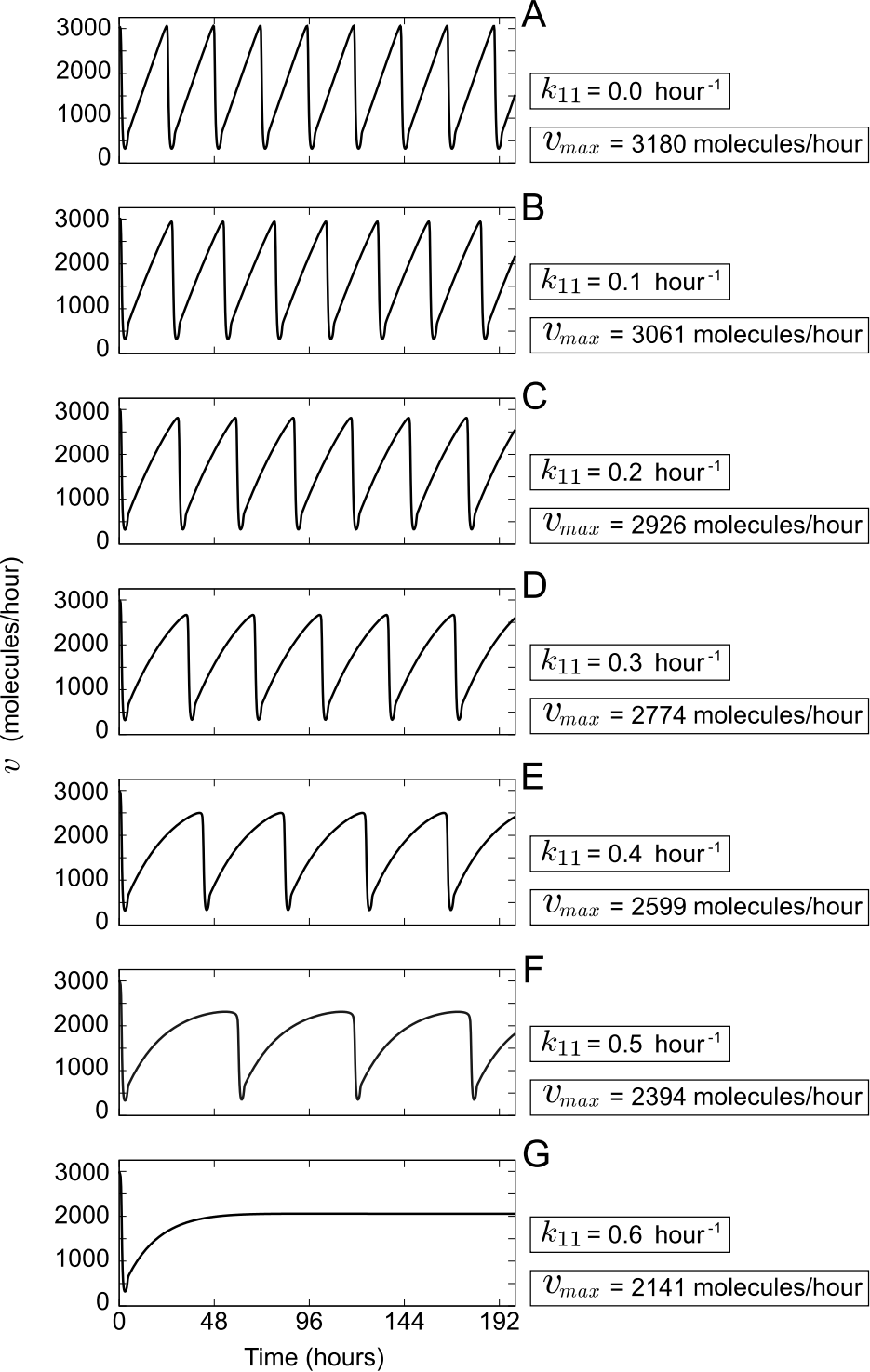

Supplement: Figure S5 — Rate of the negative interaction for different values of . Rate of the negative interaction () for different values of , where is the rate of reaction . Deterministic simulations A, B, C, D, E, F and G correspond to equals 0.0, 0.1, 0.2, 0.3, 0.4, 0.5 and 0.6 hour, respectively. The values of the other parameters are as in the section Methods: Biochemical reactions and rates. The oscillations stop when 0.6 hour (G). If is increased, increases slower, and its maximum value () is lower. The value of corresponds to the peak of the oscillations ( is the value of the steady state in G). (PDF) [file pone.0027414.s005.pdf]

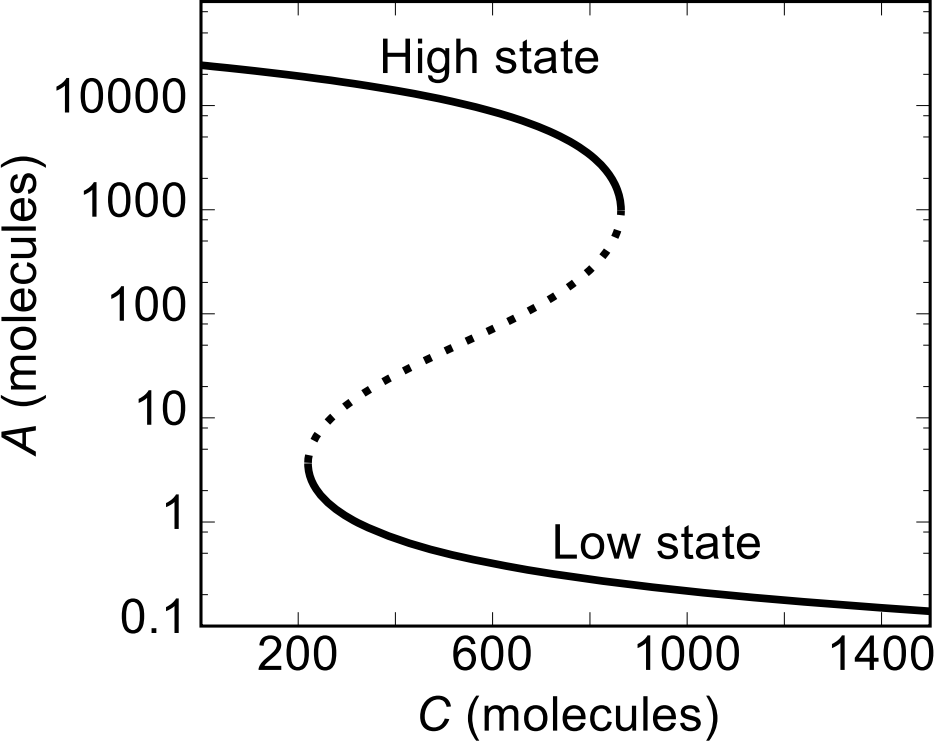

Supplement: Figure S6 — Hysteresis diagram. Hysteresis diagram depending on . The curve is the solution of the equation , where is assumed constant. The two solid lines in the diagram are the two stable steady states “high” and “low” as a function of . The dashed line represents the unstable points in the diagram. (PDF) [file pone.0027414.s006.pdf]
