# Supplementary material for: A Simple Negative Interaction in the Positive Transcriptional Feedback of a Single Gene Is Sufficient to Produce Reliable Oscillations
Source: PLoS One. 2011 Nov 10;6(11):e27414. doi: 10.1371/journal.pone.0027414 (PMC3244268; doi:10.1371/journal.pone.0027414)
Supplement: TableS1 — Data points of Fig. 4I . (PDF) [file pone.0027414.s007.pdf]

Table S1. Data points of Fig. 4I.

	Period of A		Amplitude of A	
**Changed rate**	**Mean (hours)**	**S.D. (hours)**	**Mean (molecules)**	**S.D. (molecules)**
none	24.3	1.7	6723	858
$1.15k_1$	23.9	1.8	6554	909
$0.85k_1$	24.6	1.6	6792	867
$1.15k_{-1}$	24.5	1.6	6762	822
$0.85k_{-1}$	23.9	1.9	6528	974
$1.15k_2$	23.6	1.7	6365	879
$0.85k_2$	24.8	1.7	7017	865
$1.15k_3$	27.5	1.8	8796	1042
$0.85k_3$	21.0	1.7	4760	740
$1.15k_4$	21.9	1.6	5263	731
$0.85k_4$	27.2	1.8	8714	1082
$1.15k_5$	26.9	1.8	8458	1047
$0.85k_5$	21.6	1.7	5053	712
$1.15k_6$	23.6	1.6	6247	803
$0.85k_6$	24.8	1.8	7138	1020
$1.15k_7$	24.6	1.7	6807	860
$0.85k_7$	23.9	1.8	6501	959
$1.15k_8$	21.5	1.6	5931	880
$0.85k_8$	27.8	1.8	7597	881
$1.15k_9$	21.9	1.6	6021	859
$0.85k_9$	27.3	1.8	7485	937
$1.15k_{10}$	24.3	1.6	6886	880
$0.85k_{10}$	24.3	1.7	6486	889
