# Supplementary material for: A Simple Negative Interaction in the Positive Transcriptional Feedback of a Single Gene Is Sufficient to Produce Reliable Oscillations
Source: PLoS One. 2011 Nov 10;6(11):e27414. doi: 10.1371/journal.pone.0027414 (PMC3244268; doi:10.1371/journal.pone.0027414)
Supplement: Table S2 — Data points of locus Hopf bifurcation in reduced model (Fig. S4). (PDF) [file pone.0027414.s008.pdf]

Table S2. Data points of locus Hopf bifurcation in reduced model (Fig. S4).

k_8 (hour^{-1})	k_9^{min} (molecules hour^{-1})	k_9^{max} (molecules hour^{-1})
0.1	4.65	220.3
1	4.70	219.3
2.6	4.78	217.6
3	4.80	217.1
3.5	4.82	216.6
5	4.90	214.9
10	5.17	209.6
20	5.75	199.3
30	6.39	189.7
40	7.10	180.8
80	10.5	150.4
120	14.8	127.1
160	20.1	108.4
200	26.9	91.9
225	32.7	81.5
250	41.4	68.8
255	44.2	65.3
260	48.3	60.5
261	49.6	59.1
262	51.5	57.0
262.6	53.7	54.8
