# Supplementary material for: A Simple Negative Interaction in the Positive Transcriptional Feedback of a Single Gene Is Sufficient to Produce Reliable Oscillations
Source: PLoS One. 2011 Nov 10;6(11):e27414. doi: 10.1371/journal.pone.0027414 (PMC3244268; doi:10.1371/journal.pone.0027414)
Supplement: Table S3 — Data points of locus of oscillations with less than 20% of correlation in the first period in the stochastic model (Fig. S4). (PDF) [file pone.0027414.s009.pdf]

Table S3. Data points of locus of oscillations with less than 20% of correlation in the first period in the stochastic model (Fig. S4).

k_8 (hour^{-1})	k_9^{min} (molecules hour^{-1})	k_9^{max} (molecules hour^{-1})
0.5	3	323
1	3	271
5	4	125
10	5	77
15	5	53
20	5	30
24	16	18
