# Supplementary material for: A Simple Negative Interaction in the Positive Transcriptional Feedback of a Single Gene Is Sufficient to Produce Reliable Oscillations
Source: PLoS One. 2011 Nov 10;6(11):e27414. doi: 10.1371/journal.pone.0027414 (PMC3244268; doi:10.1371/journal.pone.0027414)
Supplement: Text S1 — The negative interaction does not produce an effective high nonlinearity. (PDF) [file pone.0027414.s010.pdf]

Supporting Information: A simple negative interaction in the positive transcriptional feedback of a single gene is sufficient to produce reliable oscillations

Jesús M. Miró-Bueno*, Alfonso Rodríguez-Patón*
Departamento de Inteligencia Artificial, Facultad de Informática, Universidad Politécnica de Madrid, Madrid, Spain
∗ **E-mail: jmiro@fi.upm.es; arpaton@fi.upm.es**

The negative interaction does not produce an effective high nonlinearity

It has been demonstrated that protein sequestration produces an effective high nonlinearity [1, 2]. But this high nonlinearity is not observed if the repressor molecule is recycled (see equation S9 and figure S5 in [1]). The biochemical reactions that describe the negative interaction are as follows:

$$\begin{aligned}
&A \text{ creation (or entry):} && \xrightarrow{f} A \\
&A \text{ degradation:} && A \xrightarrow{k_6} \phi \\
&\text{Complex creation:} && R + A \xrightarrow{k_7} C \\
&\text{Complex decay into R:} && C \xrightarrow{k_8} R \\
&R \text{ creation (or entry):} && \phi \xrightarrow{k_9} R \\
&R \text{ degradation (or exit):} && R \xrightarrow{k_{10}} \phi.
\end{aligned} \tag{1}$$

The dynamics of these reactions are described by the following EDOs:

$$\begin{aligned}
dA/dt &= f - k_6 A - k_7 AR \\
dR/dt &= -k_7 AR + k_8 C + k_9 - k_{10} R \\
dC/dt &= k_7 AR - k_8 C,
\end{aligned} \tag{2}$$

As in [1], these equations can be solved at steady state to yield:

$$\begin{aligned}
A &= \frac{f k_{10}}{k_7 k_9 + k_6 k_{10}} \\
R &= \frac{k_9}{k_{10}} \\
C &= \frac{f k_7 k_9}{(k_7 k_9 + k_6 k_{10}) k_8},
\end{aligned} \tag{3}$$

where we observe no nonlinearity in output A as a function of input flux f.

References

1. Buchler NE, Louis M (2008) Molecular titration and ultrasensitivity in regulatory networks. J Mol Biol 384:1106-1119.

2. Buchler NE, Cross FR (2009) Protein sequestration generates a flexible ultrasensitive response in a genetic network. Mol Syst Biol 5:272.
